# Functional evaluation of novel compound heterozygous variants in *SLC12A3* of Gitelman syndrome

**DOI:** 10.1186/s13023-025-03577-8

**Published:** 2025-02-11

**Authors:** Na Wang, Yuanxing Yang, Xiong Tian, Hongjun Fu, Shuaishuai Chen, Juping Du, Mengyi Xu, Haixia He, Bo Shen, Jiaqin Xu

**Affiliations:** 1 Department of Clinical Laboratory, Taizhou Hospital of Zhejiang Province Affiliated to Wenzhou Medical University, 150 Ximen Street, Linhai, China; 2 Department of Endocrinology, Taizhou Hospital of Zhejiang Province Affiliated to Wenzhou Medical University, Linhai, China; 3 Department of Public Research Platform, Taizhou Hospital of Zhejiang Province Affiliated to Wenzhou Medical University, Linhai, China

**Keywords:** Gitelman syndrome, Hypokalemia, SLC12A3, Gene mutation, Loss-of-function

## Abstract

**Background:**

Gitelman syndrome (GS) is an inherited renal tubular disorder characterized by hypokalemic alkalosis and hypomagnesemia, due to biallelic pathogenic variants in the solute carrier family 12 member 3 (*SLC12A3*) gene encoding a sodium-chloride (Na-Cl) cotransporter (NCC). This work aimed at identifying *SLC12A3* variants in the GS pedigree and reveal the effect of the mutations on protein structure and function.

**Methods:**

Whole-exome sequencing (WES) and Sanger sequencing were performed in the pedigree. Configuration prediction of two mutant NCC proteins were achieved using SWISS-MODEL. The *SLC12A3* missense mutants were generated by site-specific mutagenesis, and the protein expression, location and Na^+^ uptake activity were assessed by using the HEK293T cell line.

**Results:**

Genetic analysis identified novel compound heterozygous *SLC12A3* variants (c.718G > A/p.E240K and c.2675T > C/p.L892P) in the patient with typical GS phenotype. Both of her parents, elder brother and her son carried the heterozygous p.L892P variant, but only the elder brother exhibited mild hypokalemia. Bioinformatics tools predicted that both mutations were highly species conserved and pathogenic. The prediction of mutant protein indicated that p.E240K and p.L892P altered protein’s secondary and three-dimensional (3D) structure and stability. Functional experiments revealed decreased protein expression and Na^+^ uptake activity caused by these two variants, especially the p.L892P variant.

**Conclusion:**

Our study presents the genetic and functional evidence for the novel compound heterozygous loss-of-function variants in *SLC12A3* that may synergistically cuase GS, and expands the mutation spectrum of *SLC12A3* variants in patients with GS.

## Introduction

Gitelman syndrome (GS) is an autosomal recessive renal tubular disorder with a general incidence of 1/40,000 and an estimated incidence of 3% in Chinese populations [1, 2]. Its main clinical characteristics include hypokalemic metabolic alkalosis with hypomagnesemia, hypocalciuria, normotension or hypotension [3], and most patients present with mild and nonspecific symptoms during adolescence or adulthood, such as fatigue and abdominal pains [4]. In recent years, growing evidence shows that patients with GS may suffer from growth retardation, tetany, epilepsy and rhabdomyolysis in addition to electrolyte abnormalities [5, 6], and they are also at higher risk for fatal arrhythmia, type 2 diabetes and renal failure [7–9]. Therefore, early diagnosis and active treatment are of great significance for ameliorating symptoms and disease progression for GS patients.

The genetic aetiology of GS is, in most cases, the mutations in the solute carrier family 12 member 3 (*SLC12A3*) gene on human chromosome 16q13 [10, 11]. The human SLC12A3 gene consists of 26 exons and encodes a thiazide-sensitive sodium-chloride (Na-Cl) cotransporter (NCC) harboring 12 transmembrane domains and the intracellular hydrophilic amino and carboxyl termini [12], which is specifically expressed on the luminal membrane of the distal convoluted tubules in the kidney and principally mediates the reabsorption of sodium and chloride [13]. To date, over 500 distinct *SLC12A3* mutations have been reported to be associated with GS worldwide [14, 15], and most of GS cases (up to 70%) carried the compound heterozygous mutations, while only a small percentage of the patients showed homozygous genotype and more severe clinical symptoms [16, 17]. Advances in the identification and functional characterization of mutations in GS patients using Xenopus oocytes, mammalian cell lines or mouse models increase our knowledge about human NCC function [18–20]. Therefore, a combined structural and functional analysis to characterize new *SLC12A3* variants may help uncover the underlying molecular mechanism and basis for this disorder.

In this study, we identified novel compound heterozygous *SLC12A3* variants (c.718G > A/p.E240K and c.2675T > C/p.L892P) in the GS family using whole-exome sequencing (WES). Bioinformatics prediction indicated that both mutations both mutations were highly species conserved and pathogenic, affecting the secondary structure of NCC protein. Structural modeling showed that both mutations exhibited devastating effects on the three-dimensional (3D) structure and stability of NCC protein. Besides, in vitro functional experiments revealed a notable reduction of protein expression and Na^+^ transport activity caused by these two variants, especially the p.L892P variant. Overall, our study strengthens the genetic and functional evidence for the p.E240K and p.L892P loss-of-function variants in *SLC12A3* that may synergistically cause GS.

## Materials and methods

### Sample collection and molecular testing

Peripheral blood samples were taken from the patient and her family members, and their clinical manifestations and biochemical parameters were collected for analysis. Genomic DNA extraction and genetic testing analysis were performed in DIAN DIAGNOSTICS Co. Ltd (Hangzhou, Zhejiang, China). Whole exome sequencing (WES) was applied to screen genes associated with endocrine system diseases, and the candidate variants were validated using Sanger sequencing. The identified variations and related diseases were retrieved using the ClinVar, gnomAD, ExAC or HGMD database. Variant pathogenicity and annotation were evaluated according to the classification criteria and guidelines of the American College of Medical Genetics and Genomics (ACMG) [21].

### Bioinformatics analysis

Evolutionary conservation of *SLC12A3* variants was assessed by aligning the amino acid sequences across eight species, including human, mouse, rat, rabbit, zebrafish, dog, horse and pig. The potential impacts of variants on NCC protein were estimated using online tools, including SIFT, PolyPhen-2, MutationTaster, FATHMM, DUET, SOPMA, Predictprotein and Missense3D. The predicted 3D structures of wild type (wt) and mutant NCC proteins were accessible via SWISS-MODEL (https://swissmodel.expasy.org/), and their corresponding protein configuration were visualized using PyMOL Viewer.

### Plasmid construction and cell transfection

To explore the function of the two identified variants, two distinct set of wt and mutant vectors were constructed. Full-length NCC was acquired from a human cDNA library (Bioeagle, Wuhan, China). Subsequently, the cDNA of wt NCC was separately cloned into the FLAG-tagged pcDNA3.1 vector and p3×Flag-CMV-7.1 vector (Bioeagle, Wuhan, China). The c.718G > A and c.2675T > C mutant were generated by utilizing a site-directed mutagenesis kit (TaKaRa, Japan) according to the manufacturer’s instructions. The primers were as follows: *SLC12A3*-mut1 (c.718G > A/p.E240K): forward 5′-ACG GTGGGCTTTGCAAAGACCGTGCGGGACC-3′, reverse 5′-GGTCCCGCACGGTCTTTGCAAAGCCCACCGT, reverse 5′-GGTCCCGCACGGTCTTTGCAAAGCCCACCGT-3′, *SLC12A3*-mut2 (c.2675T > C/p.L892P): forward 5′-GGCGATCATTTCTCTGCCGAGCAAGTTCCGACTGGG-3′, reverse 5′-CCCAGTCGGAACTTGCTCGGCAGAGAAATGATCGCC-3′. Successful mutagenesis was confirmed by Sanger sequencing. Human embryonic kidney (HEK) 293T cells were then transfected with all plasmids using Lipofectamine™ 2000 transfection reagent (Invitrogen, Shanghai, China) at around 80% confluency and cultured for 48 h.

### RNA extraction and quantitative real-time PCR

Total RNA was isolated using Trizol (RNAiso PLUS, TaKaRa, Japan) and reversetranscribed into cDNA using Hifair^®^ V nCov Multiplex One Step RT-qPCR Probe Kit (YEASEN, Shanghai, China). The gene expression levels of SLC12A3 were detected by BioRad T100 and normalized to actin according to the 2^−ΔΔCt^ method. Primer sequences are listed as follows: *SLC12A3*: forward 5′-CAAGGATGACGATGACAAGC-3′, reverse 5′-TCGTGTTGTAGCCAAAGGTG-3′; actin: forward 5′-CCACCATGTACCCAGGCATT-3′, reverse 5′-CGGACTCATCGTACTCCTGC-3′.

### Western blotting

For determination of the protein expression levels of wt and mutant NCC, cells at 48 h post-transfection were lysed using RIPA buffer with 1% PMSF (Beyotime, Shanghai, China). Denatured proteins were subjected to 12% SDS-PAGE separation and transferred to a polyvinylidene fluoride membrane (Millipore, Billerica, USA). Blots were then incubated with anti-SLC12A3 antibody (1: 1000, ABclonal, China) or anti-GAPDH (1: 1000, CST, USA) at 4 °C overnight, followed by probing with HRP-conjugated secondary antibody (1: 20000, DIAAN, China) for 1 h at room temperature. Enhanced chemiluminesce was then utilized for signal detection.

### Immunofluorescence staining

At 24 h post-transfection, cells were fixed with 4% paraformaldehyde, permeabilized with 0.2% Triton X-100 and blocked using 5% goat serum. Afterward, cells were washed and incubated overnight with anti-Flag (1:2000, DIAAN, China) and anti-ZO-1 (1:200, Proteintech, China), and then probed with mouse anti-488 (1:200, ABclonal, China) and rabbit anti-Cy3 (1:200, ABclonal, China) for 2 h at 37 °C, followed by DAPI staining. The images were viewed and photographed under a confocal microscope (FV-1200, Olympus, Japan).

### Na^+^ uptake assay

At 48 h post-transfection, cells were collected and seeded into 96-well plates at 5 × 10^4^ cells/well and culture medium was removed after cell adherence. 5 µM ENG-2 AM probe was added and incubated at 37 °C for 1 h, and then serum-free medium without AM probe was incubated for an additional half an hour. Fluorescence signals were finally detected using a multiscan spectrum EnVision^®^ (EnVision, PerkinElmer, USA).

### Follow-up studies

The patient was reviewed by face-to-face in our hospital every 2 ~ 3 months to record symptoms. Biochemical detection was also performed to monitor changes in blood potassium, and the occurrence of potential complications such as glucose metabolism and renal function were also closely tracked during the follow-up. The pedigree members were also reviewed by phone or face-to face to determine if they showed a phenotype of GS.

### Statistical analysis

Quantitative data was represented as means ± standard deviation (SD) and analyzed by GraphPad Prism 8.0 (Graphpad, CA, USA). Comparisons were performed with unpaired Student t-test (two-sided). *P* < 0.05 was considered statistically significant.

## Results

### Clinical features

The 40-year-old female patient was admitted to the Department of Endocrinology in our hospital due to hypokalemia for 1 year and muscle weakness along with limb numbness for 2 days. In the previous visits, she was diagnosed as hypokalemia at a local hospital with the lowest blood potassium level of 2.5 mmol/L, and her symptoms were improved after receiving oral potassium therapy. However, the patient’s condition remain unstable, and she developed limb weakness and numbness and could not walk two days before admission. Physical examination on admission demonstrated a temperature of 36.7 C, heart rate of 91 bpm per minute, respiratory rate of 18 breaths per minute and blood pressure of 118/79 mmHg. She weighed 60 kg with a height of 168 cm, and her body mass index was normal (23.4 kg/m^2^). No abnormalities were found in muscular tension, renal ultrasound, adrenal CT scan and electrocardiogram. Laboratory findings indicated that the GS subject presented with hypokalemia, metabolic alkalosis, hypomagnesemia, hypocalciuria, elevated concentrations of plasma renin activity and aldosterone (Table 1). After receiving oral administration of the 10% potassium chloride and magnesium sulfate, intravenous infusion of potassium chloride and oral spironolactone at a dose of 20 mg three times a day, her symptoms was improved and discharged from hospital five days later.

**Table 1:** Biochemical characteristics of the GS subject

Characteristics	Test value	Reference value
**Age at onset/diagnosis**	39/40	—
**SBP/DBP** (mmHg)	118/79	SBP: 90-140mmHg; DBP:60–90 mmHg
**Serum biochemical analysis**		
Potassium (mmol/L)	2.05	3.50–5.30
Magnesium (mmol/L)	0.59	0.75–1.02
Calcium (mmol/L)	2.36	2.08–2.60
Sodium (mmol/L)	127.5	137.0-147.0
Chloride (mmol/L)	84.1	99.0-110.0
Phosphorus (mmol/L)	1.07	0.96–1.34
Creatinine (µmol/L)	59	59–104
Urea nitrogen (mmol/L)	4.58	3.1-8.0
**Arterial blood gas analysis**		
PH	7.48	7.35–7.45
HCO_3_(mmol/L)	29.0	21.0–27.0
PO_2_ (mmHg)	100	80–100
PCO_2_ (mmHg)	39	35–45
**24-h urine test**		
Potassium (mmol/24 h)	84.2	25–100
Calcium (mmol/24 h)	1.43	2.5–7.5
Sodium (mmol/24 h)	126.7	130–260
Chloride (mmol/24 h)	257.6	110–250
Magnesium (mmol/24 h)	6.4	—
**Corticotropin and cortisol levels**		
Cortisol (8 am) (µg/dL)	15.0	6.7–22.6
Cortisol (4 pm) (µg/dL)	5.7	<10.0
Cortisol (24 h) (µg/dL)	1.98	<10.0
ACTH (8 am) (pg/mL)	19.4	7.2–63.3
ACTH (4 pm) (pg/mL)	7.8	3.0–30.0
ACTH (24 h) (pg/mL)	1.3	<20.0
**RAAS (lying condition)**		
Renin (µg/L/h)	2.53	0.13–1.74
Angiotensin II (ng/L)	78.7	23.0–75.0
Aldosterone (pg/mL)	258	30–160
**RAAS (standing condition)**		
Renin (ng/mL/h)	8.05	1.45-5.00
Angiotensin II (ng/L)	70.1	32.0–95.0
Aldosterone (pg/mL)	259	70–300

SBP: systolic blood pressure; DBP: diastolic blood pressure, ACTH: adrenocorticotropic hormone; RAAS: renin-angiotensin-aldosterone system; h: hour

### Identification of *SLC12A3* mutations and pathogenicity analysis

**Fig. 1:**
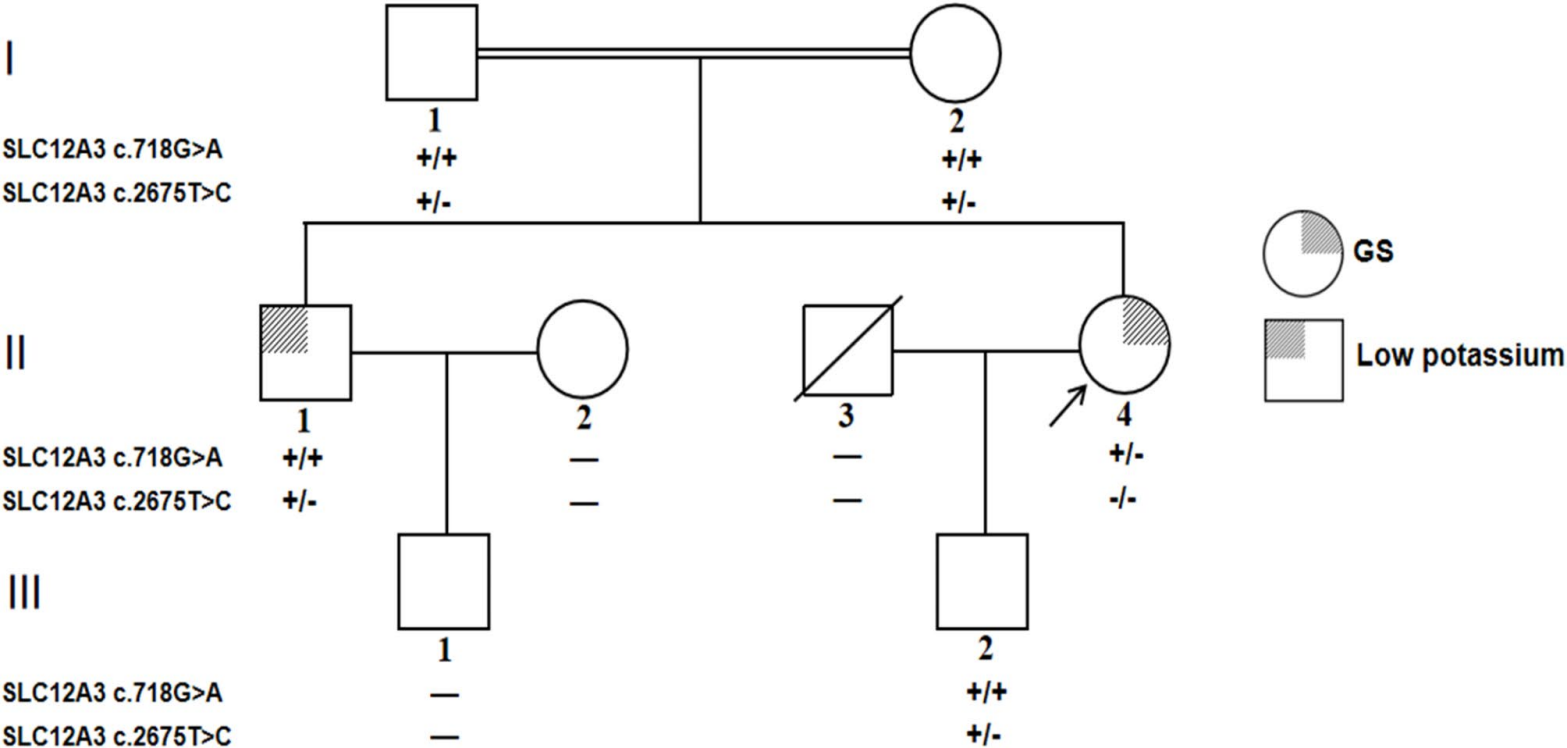
Pedigree of the GS family. The arrow points to proband (II-4). Her elder brother (III-1) harboring the heterozygous c.2675T > C/p.L892P mutation was affected by hypokalemia. Squares represent males and circles indicate females. A single diagonal line indicates deceased individuals

**Fig. 2:**
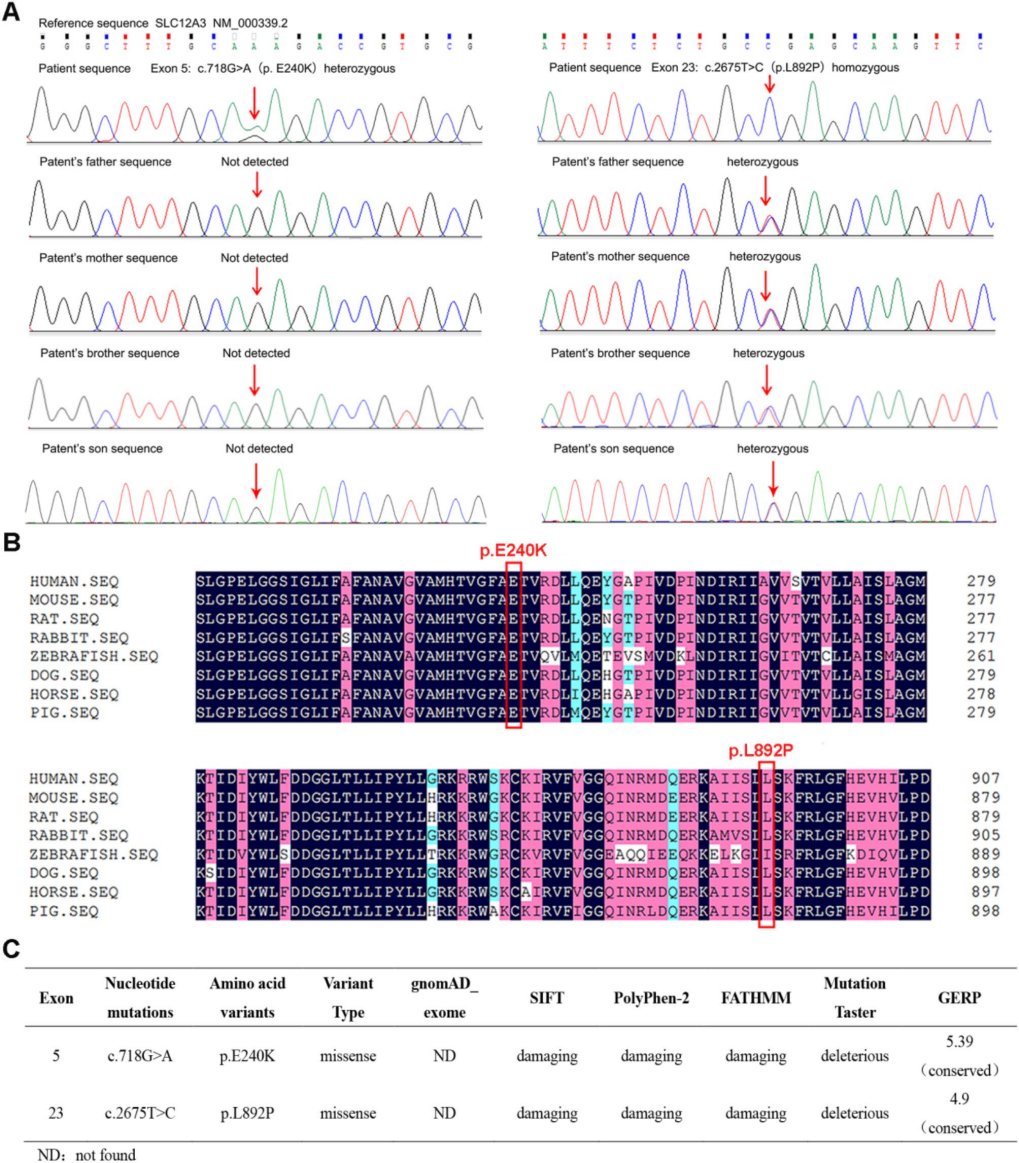
Sequencing analysis of the *SLC12A3* mutations in the pedigree. **(A)** Sanger sequencing results of *SLC12A3* variants, and arrows indicate the site of mutations. **(B)** Cross-species conservation analysis of SLC12A3 around E240K and L892P. **(C)** The pathogenicity prediction of two *SLC12A3* variants

To determine the clinical diagnosis of the proband (II-4) in this pedigree (Fig. 1). By WES analysis, we identified novel compound heterozygous variants: a new heterozygous mutation c.718G > A/ p.E240K in exon 5 and a rare homozygous mutation c.2675T > C/p.L892P in exon 23 of the *SLC12A3* gene. Sanger sequencing confirmed that the c.718G > A variant was not detected in the patient’s parents, and her father (I-1), mother (I-2), elder brother (II-1) and her son (III-1) were all carried the heterozygous c.2675T > C variant (Fig. 2A). Conservative analysis showed that the glutamate in residue 240 and the leucine in residue 892 of the NCC protein were both highly conserved among species (Fig. 2B), and both variants were strongly predicted to be damaging or deleterious by multiple prediction programs, including MutationTaster, PolyPhen-2, SIFT and FATHMM (Fig. 2C). Besides, the c.718G > A variant has not been reported previously or included in the the human gene mutation database (HGMD), while the c.2675T > C variant has been included in the HGMD database. According to the ACMG criteria, the novel c.817G > A/p.E240K mutation in *SLC12A3* was classified as uncertain significance. This mutation was a *de novo* variant that have not been validated in parental samples (PM6) and it was not found in the normal population in ESP, 1000 genomes and ExAC databases (PM2_PP), which was also pathogenic as predicted by multiple programs (PP3). The c.2675T > C/p.L892P mutation was also classified as uncertain significance based on ACMG guidelines. This variant had been reported in Taiwanese patients with GS (PS4_PP) [17, 22]. Moreover, it was located in the hot mutation region, namely, the intracellular C-terminal domain of NCC (PM1) [23], and was not found in the normal population database (PM2_PP), which was also predicted to be pathogenic by multiple programs (PP3).

### Impacts of *SLC12A3* mutations on the secondary and 3D structure of NCC protein

**Fig. 3:**
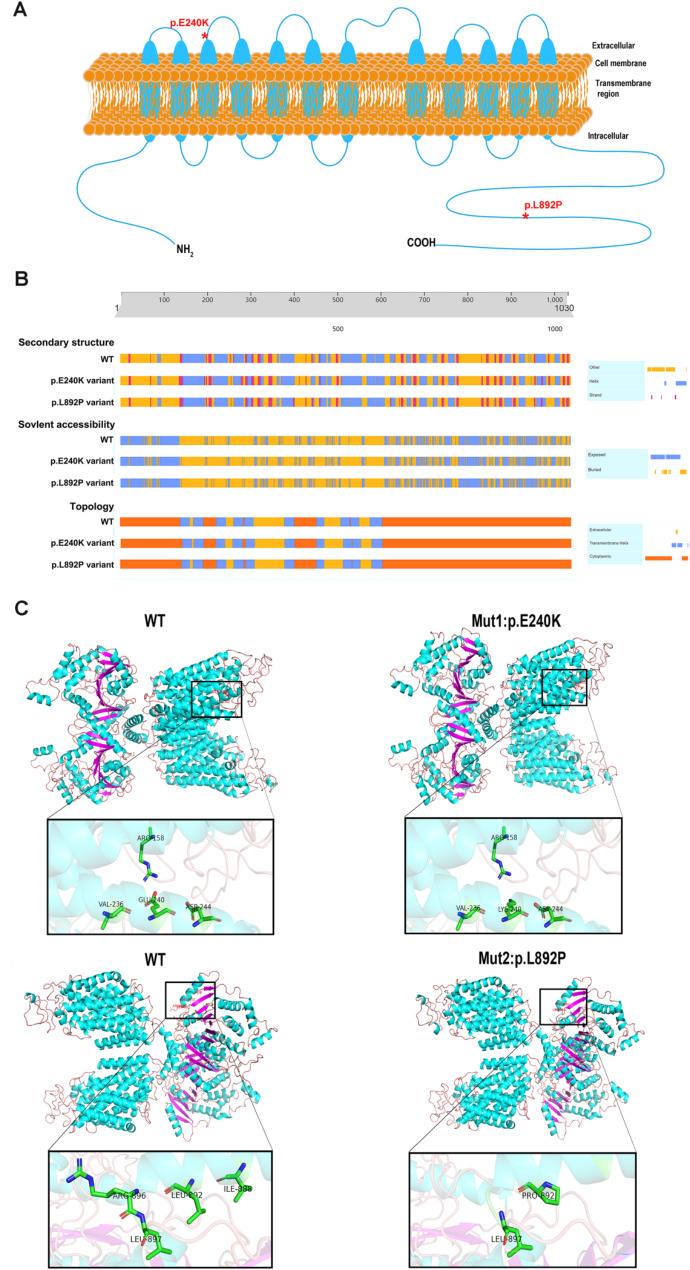
Effects of *SLC12A3* variants on the protein structure. **(A)** Schematic topological representation of NCC, and the predicted location of mutations found in our study were denoted by red asterisk. **(B)** The alterations of secondary structure and solvent accessibility of NCC by PredictProtein online database analysis. **(C)** The alterations of the 3D-structure of NCC proteins by SWISS-MODEL analysis

We next sought to explore the effects of two mutations on the structure of NCC protein. The localization of each mutation on the predicted topology of NCC was shown in Fig. 3A. The p.E240K variant was positioned on the extracellular edge of the third transmembrane segment of NCC protein, while the p.L892P variant was located in the intracellular carboxyterminal domain. Both mutations were predicted to alter the proportion and position of alpha helix, extended strand and random coil in the secondary structure of NCC protein using SOPMA database, and altered the solvent accessibility and topology of NCC protein compared to wt NCC (Fig. 3B). Structural modeling revealed that the p.E240K mutation altered the distance at which the backbone GLU240 formed hydrogen bonds with VAL236 and ASP244, resulting in a new hydrogen bond between LYS240 and ARG243 in the backbone, and the original side chain also disappeared, suggesting this mutation might affect the side chain as well as the main protein chain. The p.L892P mutation broke the hydrogen bond formed by backbone LEU892 with ARG896, LEU897 and ILE888, and a new hydrogen bond was generated between backbone PRO892 and LEU897 (Fig. 3C), suggesting its potential influence on the backbone structure of NCC. Besides, Missense3D and DUET databases predicted that both mutations exhibited a destructive effect on 3D structure and structural stability of NCC, mainly by altering buried/exposed switch. These findings demonstrated that the residue of the amino acid at position 240 and 892 might be of functional importance.

### SLC12A3 variants impaired the expression and Na^+^ uptake activity of NCC protein

**Fig. 4:**
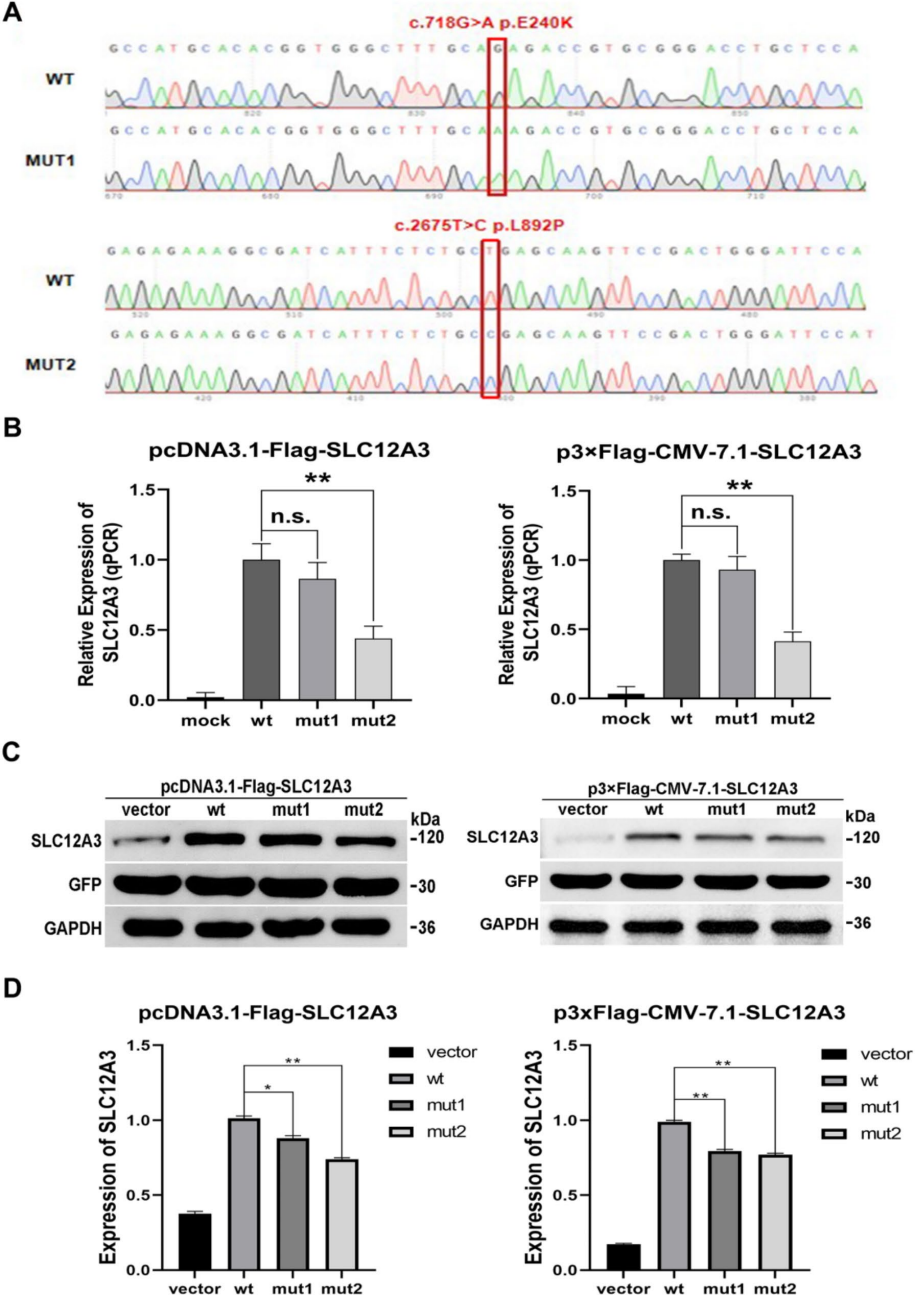
Impaired SLC12A3 expression by two mutations. **(A)** Sanger sequencing analysis confirmed the successful construction of the SLC12A3 mutant recombinant plasmids (mut1: c.718G > A/p.E240K and mut2: c.2675T > C/p.L892P). **(B)** The qRT-PCR results of the gene expression of wt and mutants. **(C, D)** The western blot results of the protein expression of wt and mutants. A significant difference was represented by * *P* < 0.05, ** *P* < 0.01, while n.s. indicates no significant difference

**Fig. 5:**
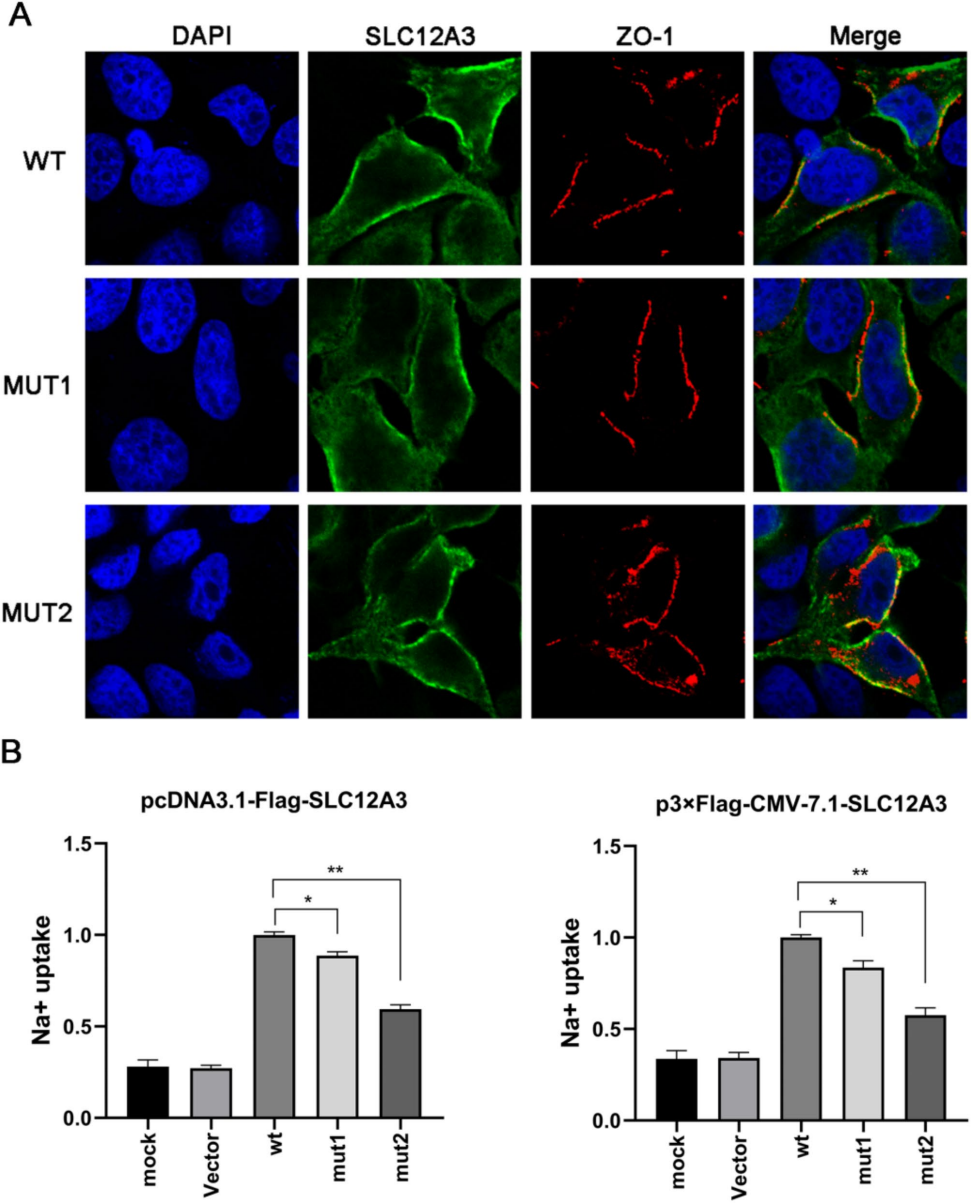
Effects of two *SLC12A3* mutations on cellular localization and Na^+^ uptake activity of NCC protein. **(A)** Immunofluorescence images showing the subcellular localization of wt and mutant NCC. **(B)** Comparison of Na^+^ uptake levels between wt and mutant NCC proteins. * *P* < 0.05, ** *P* < 0.01

To investigate the functional impacts of two *SLC12A3* mutations, we successfully constructed the *SLC12A3* mutant recombinant plasmids confirmed by Sanger sequencing (Fig. 4A), whose transfection efficiency was relatively consistent with wt plasmids significantly declined expression of *SLC12A3* mRNA observed in cells expressing p.L892P (Fig. 4B and C). Western blot analysis showed markedly decreased expression of two mutant NCC proteins in comparison to wt NCC (Fig. 4D and E), while the plasma membrane fluorescence intensity was comparable between wt and mutant NCC proteins (Fig. 5A). The impacts of two *SLC12A3* variants on the Na^**+**^ uptake activity of NCC protein were next investigated, showing that both variants significantly declined the Na^+^ uptake levels of NCC protein, with the p.L892P variant exhibiting more severe effects (Fig. 5B).

### Follow-up management

**Fig. 6:**
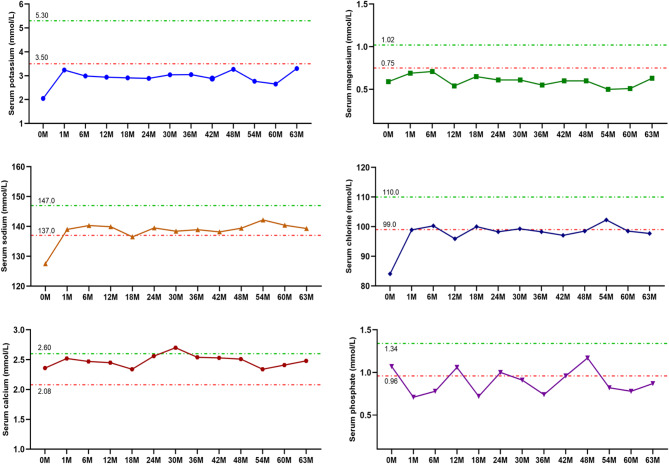
Follow-up of the biochemical indexes of the patient in our hospital, including serum potassium, magnesium, chlorine, sodium, calcium and phosphate. 0 M represents before treatment. 1 M, 6 M, 12 M, 18 M, 24 M, 30 M, 36 M, 42 M, 48 M, 54 M, 60 M and 63 M represent after treatment. M indicates month

Of note, we have also been monitoring the disease progression of the patient closely, and the follow-up interval is 2 ~ 3 months. During the follow-up of nearly 5 years, the patient took medication regularly, including oral potassium chloride sustained release tablets at the dose of 1.0 g three times a day, calcium and magnesium tablets at the dose of 1.0 g three times a day and spironolactone at the dose of 20 mg three times a day. Her serum potassium and magnesium were still below the normal range after treatment, while serum sodium and chlorine levels almost returned to normal (Fig. 6). Her overall condition remained stable throughout the follow-up period, despite occasional limb weakness.

## Discussion

In the present study, two rare mutations of the SLC12A3 gene, c.718G > A/p.E240K and c.2675T > C/p.L892P, were identified in the female patient with typical clinical phenotype of GS. However, there is a lack of functional studies on these two mutations. Here, we reported the clinical description of the female proband in a Chinese family, confirming the disease phenotype, and further assessed the pathogenicity and structural alternations of two mutations through in silico algorithms and structural modeling. In vitro functional experiments were additionally conducted in transiently transfected HEK293T cells, including NCC protein expression, cellular localization, and transport of Na^+^. The results of our study demonstrated that both variants exhibited reduced protein expression, altered protein structure, and protein dysfunction validated by Na^+^ uptake experiment.

Currently, 570 different *SLC12A3* mutations associated with GS have been included in HGMD (Professional 2023.04), with T60M, D486N, and R928C identified as common *SLC12A3* mutations in the Chinese patients with GS [15, 24]. However, none of these three mutations was detected in our research. WES analysis revealed a novel variant p.E240K and a rare homozygous p.L892P in the GS patient. Bioinformatic analysis showed that both mutations were highly conserved among multiple species and predicted to be damaging or deleterious, causing the changes of the solvent accessibility and secondary structure of NCC protein. Solvent accessibility is a critical characteristic that determines protein folding and stability, and most of disease-related single residue variations occur in buried positions [25]. Consistently, configuration prediction also revealed the devastating impacts of both mutations on the protein’s 3D structure and stability mainly by affecting buried/exposed switches. Notably, the new p.E240K variant was located nearby the third large extracellular loop, suggesting that this amino acid might play a role in ion affinity [4]. The second mutation (p.L892P) was located within the intracellular C-terminal domain, which was considered as a hot spot for mutations and this region included three putative protein kinase phosphorylation sites that might affect the NCC activity and a binding domain of a protein disulfide isomerase that affects protein folding [23], indicating that the amino acid at 892 position might be important for maintaining the activity of NCC. However, whether the two mutations affect protein expression and activity and thus the final GS phenotype remains to be elucidated.

It is well known that different types of point mutations in the same gene may exert distinct effects on protein biological processes, such as translation, splicing or protein activity [26]. Thus, determining the impact of mutations on protein biological functions is crucial for etiological mechanism investigation. Previous studies suggested that a number of *SLC12A3* missense mutations causing GS could cause impaired NCC expression and/or activity [20, 27]. The potential molecular mechanisms included impairing protein synthesis, the failure of functional protein insertion into the cell membrane because of the lack of glycosylation, impairing protein processing resulting in its retention and degradation in endoplasmic reticulum, the capability of reaching the cell membrane but with reduced Na^+^ uptake or accelerating the removal or degradation of proteins [5, 28, 29]. A recent study revealed that a homozygous pathogenic p.N958K mutation of *SLC12A3* could also impair the NCC function by deteriorating endoplasmic reticulum stress [19]. In our study, we performed in vitro experiments using HEK293T cells to simulate the functional effects of the variant alleles, and results indicated that both mutations significantly reduced the expression of NCC protein, although the surface localization of mutant NCC proteins was not affected. Importantly, both mutants failed to mediate the normal Na^+^ uptake of NCC compared to wt NCC. Of note, the trends in the protein expression levels of the E240K-NCC protein were similar to its observed functional levels, indicating that the declined transporter activity of NCC caused by p.E240K variant was probably attributable to its decreased protein expression. Additionally, the declined trends in the Na^+^ uptake rate of the L892P-NCC protein exhibited an even more prominent difference than its protein expression levels, revealing that the decrease in sodium intake caused by the p.L892P mutant was likely due to both the decrease of NCC expression and the transporter dysfunction. Next, we adjusted the original ACMG classification results of the two SLC12A3 mutations based on the results of in vitro functional experiments. Based on ACMG, the c.817G > A/p.E240K mutation was identified as a likely pathogenic mutation (PS3 + PM2_PP + PM6), and the c.2675T > C/p.L892P mutation was also identified as a likely pathogenic mutation (PS3 + PM1 + PM2_PP + PS4_PP + PP3).

Although the long-term prognosis of GS is favorable, the majority of patients struggle to maintain normal serum potassium levels, and chronic disorders of serum potassium and magnesium may lead to abnormal glucose metabolism or impaired renal function [30, 31]. Thus, we conducted long-time follow-up with the patient for minor therapeutic effects and disease control. The patient’s condition remained relatively stable except for occasional muscle weakness. Like most of GS patients, the patient never achieved normal levels of serum potassium and magnesium after treatment, indicating the difficulty to correct hypokalemia and hypomagnesemia in GS [32]. Remarkably, a review of animal and human research proposed the possible existence of a weak intermediate phenotype in *SLC12A3* heterozygotes [33], and a study by Wan et al., also characterized the close link between lower potassium levels and a heterozygous pathogenic variant (p.R642G) in *SLC12A3* causing GS [34]. Given these findings, we also evaluated the clinical data of four related family members carrying the p.L892 pathogenic heterozygous mutation. Her both parents and her son did not develop GS-related symptoms, while the elder brother suffered from mild hypokalemia (3.26 ~ 3.45 mmol/L) and fatigue that were relieved after receiving oral potassium chloride sustained release tablets, which may support the previous findings of intermediate phenotypes in *SLC12A3* pathogenic heterozygotes. Nevertheless, we also could not rule out the possibility of the existence of other SLC12A3 variant in her elder brother, as a recent study identified a second likely pathogenic/pathogenic SLC12A3 variant using long-read sequencing from 67 individuals with suspected GS that had only one mutant allele [35]. Further evaluation and genetic analysis of the patient’s elder brother are still warranted.

In conclusion, the present study identified a GS patient who carried novel compound heterozygous loss-of-function variants in *SLC12A3*, namely, p.E240K and p.L892P. Both mutations caused protein structure alternation and protein dysfunction validated by in vitro functional experiments, and were identified as likely pathogenic. Our study expands the mutation spectrum of the *SLC12A3* gene in patients with GS, and also provides functional evidence for evaluating the pathogenicity of the two mutations.

## Data Availability

The data presented in this study are available on request. The data are not publicly available due to privacy or ethical restrictions.

## Abbreviations

GS: Gitelman syndrome
SLC12A3: Solute carrier family 12 member 3
Na: Sodium
Cl: Chloride
NCC: Sodium-chloride cotransporter
WES: Whole-exome sequencing
3D: Three-dimensional
Wt: Wild type
CT: Computed tomography
SBP: Systolic blood pressure
DBP: Diastolic blood pressure
ACTH: Adrenocorticotropic hormone
RAAS: Renin-angiotensin-aldosterone system
ACMG: American College of Medical Genetics and Genomics
HGMD: Human Gene Mutation Database
